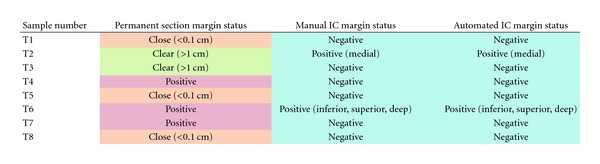# Computerized Decision Support System for Intraoperative Analysis of Margin Status in Breast Conservation Therapy

**DOI:** 10.5402/2012/546721

**Published:** 2012-11-25

**Authors:** Manuel E. Ruidíaz, Sarah L. Blair, Andrew C. Kummel, Jessica Wang-Rodriguez

**Affiliations:** ^1^Department of Bioengineering, University of California San Diego, La Jolla, CA 92093, USA; ^2^Moores Cancer Center, University of California San Diego, La Jolla, CA 92093, USA; ^3^Department of Surgery, University of California San Diego, La Jolla, CA 92093, USA; ^4^Department of Chemistry and Biochemistry, University of California San Diego, La Jolla, CA 92093, USA; ^5^Department of Pathology, University of California San Diego, La Jolla, CA 92093, USA

## Abstract

*Background.* Breast conservation therapy (BCT) is the standard treatment for breast cancer; however, 32–63% of procedures have a positive margin leading to secondary procedures. The standard of care to evaluate surgical margins is based on permanent section. Imprint cytology (IC) has been used to evaluate surgical samples but is limited by excessive cauterization thus requiring experienced cytopathologist for interpretation. An automated image screening process has been developed to detect cancerous cells from IC on cauterized margins. *Methods.* IC was prospectively performed on margins during lumpectomy operations for breast cancer in addition to permanent section on 127 patients. An 8-slide training subset and 8-slide testing subset were culled. H&E IC automated analysis, based on linear discriminant analysis, was compared to manual pathologist interpretation. *Results.* The most important descriptors, from highest to lowest performance, are nucleus color (23%), cytoplasm color (15%), shape (12%), grey intensity (9%), and local area (5%). There was 100% agreement between automated and manual interpretation of IC slides. *Conclusion.* Although limited by IC sampling variability, an automated system for accurate IC cancer cell identification system is demonstrated, with high correlation to manual analysis, even in the face of cauterization effects which supplement permanent section analysis.

## 1. Introduction

Approximately 32–63% of breast conservation therapy surgeries result in positive margins [1]. To control local recurrence of disease, a negative margin status is required [2–5]. Therefore, a positive margin diagnosis may result in multiple reexcision surgeries, increased likelihood of complications, physical discomfort, emotional distress, and decreased cosmetic outcomes [1, 6]. Several techniques have been tested for the manual intraoperative evaluation of margins including gross examination [6–8], intraoperative ultrasound [8–12], frozen section analysis (FSA) [13–17], and imprint cytology (IC) [5, 18–21]. Herein we report on the feasibility of using automated IC analysis as a margin evaluation tool.

IC is an alternative intraoperative technique to detect cancer cells at the margin surface. Following tumor excision, the sample surface is imprinted on glass slides while keeping track of the margin location [21, 22]. Cells on the margin surface are removed during imprinting by electrostatic and hydrophobic interactions with the glass slide which preferentially adsorbs epithelial and blood cells over adipose tissue [23]. The advantage of this technique is the ability to sample a large area directly from the margin surface. Additionally, since cancer cells tend to lose their intercellular adhesion properties, a further enrichment in the preferential detachment of those cell types from the tumor microenvironment during the imprint process is possible [24–26]. Any cells that adhere to the slide surface are subsequently stained and analyzed by a cytopathologist [3, 18]. IC is a much simpler and less labor intensive technique than FSA to assess margins intraoperatively. However, detecting tumor cells close to, but not at, margins is poor due to the inability to consistently sample cells below the excision surface [23]. 

Furthermore, the imprinting process as well as the cauterization during tumor excision tends to introduce staining and drying artifacts depending on environmental and sample conditions thus rending interpretation difficult [22, 27]. For these reasons, it is the least used method to assess intraoperative margins in breast conservation surgery. 

Intraoperative margin evaluation reduces reexcision rates [7, 12, 28], but none are typically employed on a routine basis because of difficulties in consistent sample preparation and analysis with modestly skilled reader input. Of the aforementioned techniques, imprint cytology is the only technique that has the potential to accurately sample the entire margin surface without affecting downstream final margin analysis of permanent sections. Additionally, the potential for automation makes IC an attractive intraoperative assessment technique.

The challenge of the imprinting process is that it creates a specimen that may be covered by epithelial cells (including cancer cells if present), erythrocytes, leukocytes, cellular debris (organelles and cellular substructures), scrape debris, cauterized debris, and staining and drying artifacts such as bubbles, stained noncellular tissue, and so forth. A standard microscope slide (25 mm × 50 mm) is sufficient to imprint a margin surface. A reasonably sized excision tissue with 6 separate margin surfaces (anterior, posterior, superior, inferior, medial, and lateral) would therefore require the manual search of 7500 mm^2^ (25 mm × 50 mm × 6) of slide area per tissue excision which is immensely time consuming and impractical for real time accurate interpretation in intraoperative settings. Furthermore, because of the sparse nature of the imprint without any of the original architectural information, interpretation based purely on cytological features is required at modest to high magnification level (10x, 20x, or 40x) by a highly trained cytopathologist to identify cancerous cells from other types of debris and artifacts. For a standard field of view of 1.4 mm by 1.0 mm (10x magnification), it is expected that 5357 fields of view must be thoroughly inspected to accurately determine margin status.

To overcome these limitations, an automated analysis has been developed to detect and identify positive cancer cells at the margin via IC. Current slide scanning technology has progressed to being able to quickly scan through slides. Additionally, the sparse nature of the IC surface implies that parallel image processing would be exploitable to analyze the entire slide. This enables the use of modern multiprocessor computing architectures to process the slides with reduced processing time in intraoperative margin assessment. Furthermore, the same technology could be employed with minor modification for core biopsy analysis or even permanent section analysis where the issues of cellular debris are much reduced.

This paper reports upon the development and evaluation of an automated imprint cytology analysis tool using retrospectively collected samples to provide directly comparable performance characteristics of automated assist versus manual techniques. Experimental methods were designed to provide results which are easily transferable and applicable to an intraoperative setting without disrupting accepted surgical and pathologic protocols. This included (1) working with primary human breast tissue, (2) excision of tumors using standard electrosurgical techniques, and (3) measurement of system performance using true cauterized margins. It is noted that the most successful study of manual margin interpretation by IC employed uncauterized margins [18]. However, accepted surgical protocols typically employ electrosurgical tools which greatly increase the amount of debris and artifacts on IC slides. This creates a more difficult and time consuming manual inspection requirement and thereby increases the need for an automated approach to IC evaluation.

## 2. Methods

Imprint cytology specimen collection was approved by the Institutional Review Board of the University of California, San Diego and was performed in accordance with all accepted standards for human clinical research. All patients gave written informed consent. A retrospective analysis of 127 patients for whom IC slide was prepared on their excised tumors was performed to identify specimens with positive or close permanent section diagnosis. 16 positive/close margin cases were selected and an 8 slide training subset was culled which consisted of 4 slides with positive margin determination and 4 slides with close margin determination (Table 1). An additional testing set was created consisting of 8 cases (exclusive of the training set) from the 16 positive/close margin selection (Table 1).

Multiple 1-inch × 3-inch glass microscope slides coated with poly-L-lysine (PLL) (Newcomer Supply, Middleton, WI) were used for imprint cytology. Six (6) margin faces (superficial, deep, superior, inferior, lateral, medial) oriented by suture placement during initial excision were used for imprint cytology. A no.10 scalpel blade (VWR International, LLC, West Chester, PA) was placed perpendicular to the tumor surface and dragged across the tissue in a scraping fashion to remove surface cautery artifact. Immediately following scraping, a PLL-coated slide was pressed onto the scraped margin face for 10 seconds and immediately fixed in pure ethanol for 24 hours, after which the slide was stained with Hæmatoxylin and Eosin (H&E).

Whole imprint cytology slides were digitized on a ScanScope XT slide scanner (Aperio Technologies, Inc., Vista, CA) at 40x (0.25 *μ*m/pixel) and saved as SVS files with JPEG 2000 compression. An IC image processing system was developed based on ImageJ [29] and modified to function on whole-slide images of H&E stained slides. 

Image analysis was done in two phases: (1) an outlining phase in which raw pixel data was converted to objects representing nuclei, debris, and artifacts; (2) an object analysis phase in which individual objects were identified and quantified across the entire slide. The software was written and designed to learn and identify cancer cells and cancer cell clusters. Training was performed on a representative set of labeled slides selected by the authors (M. E. Ruidíaz and J. Wang-Rodriguez) which contained known tumor cell clusters, debris, adipose tissue, and white blood cells. After optimizing for cancer cells, the software algorithm was tested on unlabeled samples as a measure of system performance. 

An outlining algorithm was developed and optimized for the detection of nucleated objects from cauterized and scraped imprint cytology specimens. Briefly: Each scanned whole-slide IC slide image was processed in 2000 × 2000 pixel subtitle increments with 100 pixel overlap. Each subtitle was processed in 6 serial steps: (1) Red channel filtering: mainly to remove red blood cells from the image. (2) Laplacian of Gaussian thresholding: identify object boundaries on the image. (3) Binary shape corrections: reduce noise, smooth out and improve object boundaries. (4) Watershed filtering: separate falsely concatenated nuclei. (5) Object selection: discard objects ≤10 *μ*m^2^ and ≥360 *μ*m^2^ to remove noncell like objects. (6) Object outlining: store object location and outline for measurement phase.

Individual objects were measured for 179 characteristics in the following categories. (1) Grey descriptors: grey scale intensity and texture features of the imaged object. (2) Shape descriptors: size and dimensional characteristics of outline shape. (3) Intensity and texture features within (nuclear) each object in two color models (RGB and YUV). (4) Intensity and texture features in the area immediately outside (cytoplasm) each object in RGB and YUV.

Statistical analysis was performed using the R statistical computing environment version 2.13.0. Raw values were examined to elicit sources of maximal variance for data classification studies. Multivariate linear discriminant analysis (MASS package), was used to create the training set for IC object classification. Recursive feature elimination was performed with the caret package. For error estimation, the errorest function was used in the ipred package. Cross validation was used to provide estimates of variable performance for classification and for the determination of which variables provided the greatest prognostic significance.

## 3. Results

### 3.1. Classifier Training

**Figure 1:**
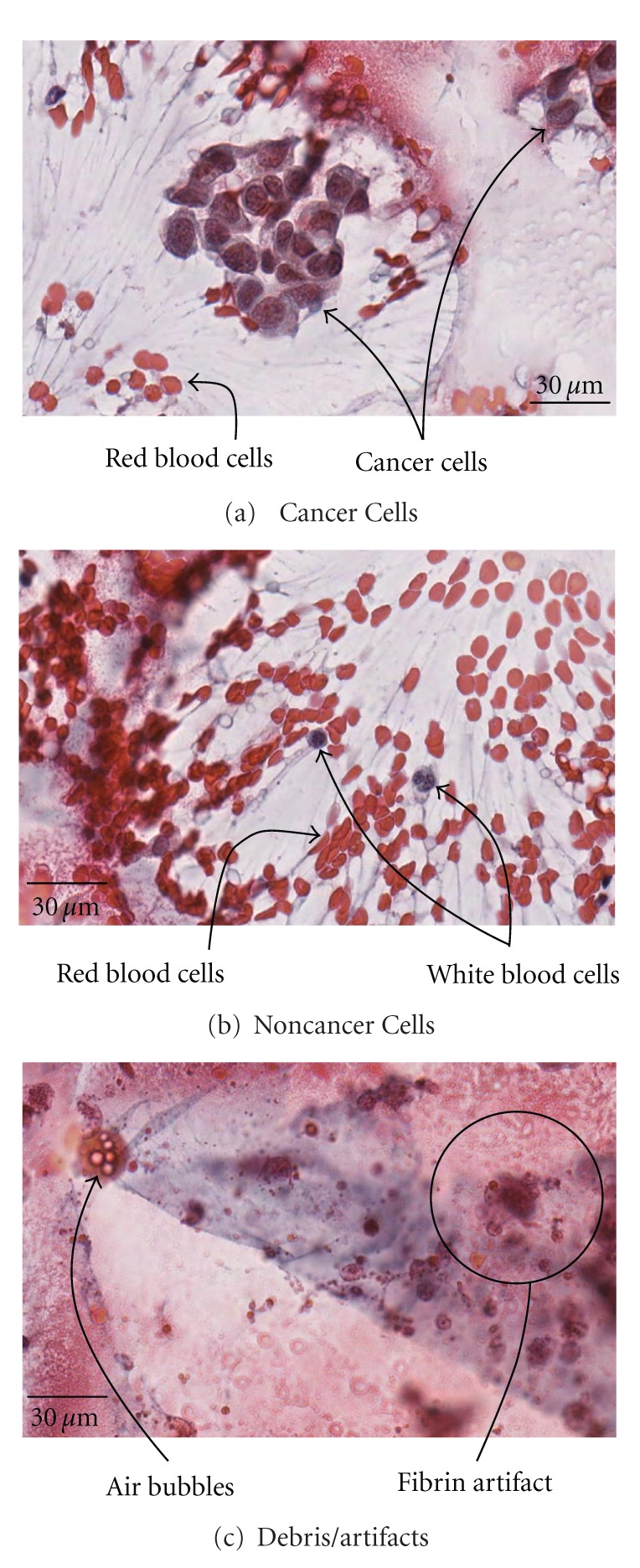
Representative imprint cytology specimens. (a) Clustered cancer cells with associated red blood cells. (b) Noncancerous individual white blood cells. (c) Debris/artifacts. Cancer cells are distinguished from other objects by their round shape, large size, staining color of the nucleus and cytoplasm, and their grouping in clusters.

**Figure 2:**
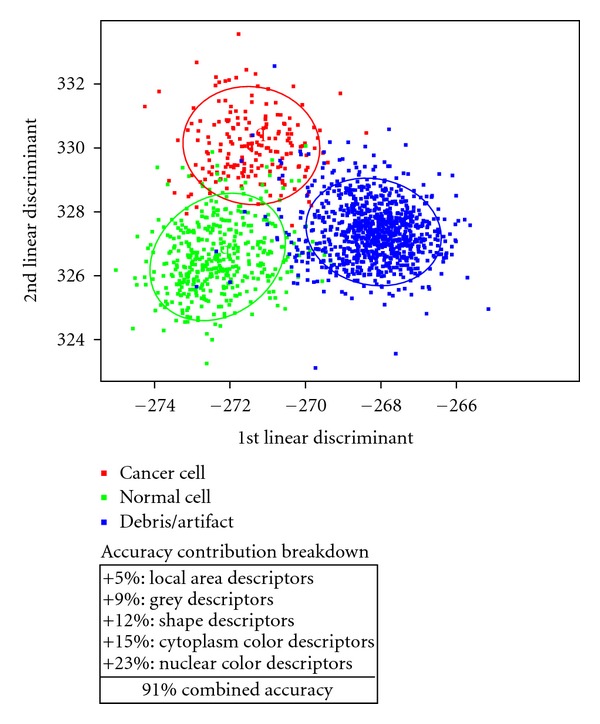
Class separation in LDA-transformed space showing cancer (red), Noncancer cells (Green), and debris/artifact objects (Blue) clustered separately. 80% normal probability ellipses for each class are overlaid. Relative contributions by class of descriptor to class separation are listed. Nuclear color provides the best discrimination.

Classifier training was performed on the 8-slide dataset as previously described (Table 1). A manual search was performed on the digitized slides, and selected objects were identified as belonging to one of three possible classes: cancer, noncancer cells, and debris/artifact (Figure 1). 184 cancer (from slides A1, A3, A5, A8), 416 noncancer (A1-A8), and 893 debris/artifact objects (A1-A8) were found manually (Table 1). It is noted that the presence of cancer cells from both positive and close margins is consistent with IC of cauterized margins sometimes being able to detect even close margins and illustrates the problem of permanent section analysis in detecting positive and close margins most likely due to sectioning limitations. This set of manually identified objects was used as the training set for a Linear Discriminant Analysis (LDA) classifier which yielded two linear discriminants, LD1 and LD2 each having a proportion of trace, a measure of how much of the between-class variance is explained, of 78% and 22%, respectively. The LDA-transformed representation of the training set is plotted in Figure 2. LDA classifier performance was analyzed with a leave-one-out cross validation yielding an overall accuracy of 91%. Individual class performance was 84%, 87% and 94% for cancer, noncancer, and debris/artifact objects, respectively.

### 3.2. Performance by Class of Descriptors and Individual Descriptor Used

To evaluate the respective contributions of each set of descriptors to overall classifier performance, classification accuracy was surveyed by independently including sets of descriptors (grey, shape, local area, nuclear color, and cytoplasm color) to train an LDA classifier based on the previously described classes. Additionally, worse case (randomized data) classification and full (combined descriptors) classification performance was evaluated. Accuracy was estimated by a 10-fold cross validation and relative accuracy performance improvements are displayed in Figure 2. Because these slides were derived from true cauterized margins (in contrast to many previous studies), a disproportionate selection of debris/artifact objects over other class types occurs. Therefore, LDA performance (10-fold cross validation) resulted in a minimum accuracy (obtained by classifying random data) of 60% and a maximum accuracy (all descriptors) of 91%. Leave-one-out cross validation indicates a high degree of discrimination (>84% accuracy) between each class which is further confirmed by the discrimination plot separation (Figure 2). 10-fold cross validation was employed to determine the characteristics which lead to the highest degree of LDA classification.

To further elucidate which individual measurements provided the greatest discrimination, an analysis of the respective contribution to the overall accuracy of the classifier was performed by evaluation of each class as a binary classification by recursive feature elimination. The variable importance in decreasing order for each class is as follows: cancer (blue-channel integrated density, U-channel integrated density, grey-channel integrated density, Y-channel integrated density, area), noncancer cell (red-channel mean intensity, red-channel median intensity, red-channel minimum intensity, U-channel maximum intensity, red-channel outer region mean intensity), debris/artifact (red-channel median intensity, red-channel mean intensity, red-channel minimum intensity, U-channel maximum intensity, U-channel integrated density).

### 3.3. System Validation on Testing Set

**Figure 3:**
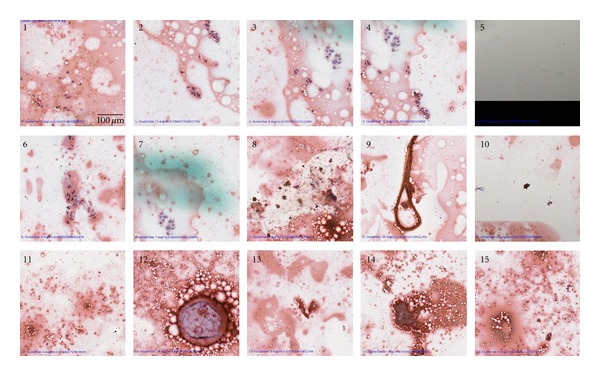
Top 15 (from 25) automatically identified suspicious cancer areas from sample T2. Cancer cells are present on areas 1, 2, 3, 4, 6, and 7. Additional misidentified noncancer objects were selected: imaging artifact at edge of slide (area 5), cautery debris (8, 9, 14), adipose (12, 14), and air bubble artifacts (8, 11, 12, 14, 15). Note: blue markings on area 7 are from manual evaluation and had no effect on automated slide evaluation.

**Figure 4:**
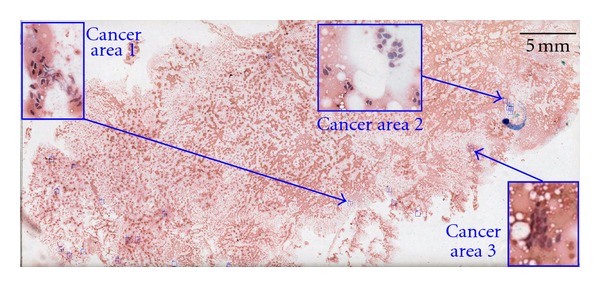
Identification of cancerous clusters across representative imprint cytology specimen T2. Cancer area 2 was determined by both automated and manual imprint cytology. Two additional foci of cancer (Cancer area 1 and 3) were additionally identified by automated imprint cytology evaluation.

Validation of the analysis system was performed using an 8-case testing subset, listed in Table 1, consisting of approximately 6 margin faces per case (46 IC specimens). The LDA classifier, created from the training set, was used to identify individual cancer cells followed by presentation of the top 25 candidate cancer clusters (≥5 cancer cells in order of decreasing average circularity) as described earlier. The performance of the top 25 selection was quantified by comparison to manual interpretation performed by JWR (Table 2). The presence of cancer cells in the top 25 cancer areas (determined automatically) was used as a measure of system performance (Figure 3). Based on retrospective samples independent from the original training data resulted in 100% agreement between an automated approach and manual interpretation illustrating the utility this approach for intraoperative analysis (Table 2). For testing set positive margins as determined by permanent section analysis (T4, T6, T7), cancer cells were only detected in 1 specimen (T6). Conversely, one sample judged clear by permanent section analysis (T2) the sampling showed signs of cancer cells via both manual and automated IC analysis (Figure 4). Additionally, two close margin training set IC specimens (A5, A8) had cancer cells, consistent with IC being able to detect true positive margins that may be erroneously called close margins due to inadequate permanent section analysis. The presence of cancer cells at the margin interface as determined by IC can only occur in the case of a positive margin status and consequently indicates that permanent section analysis may underreport true positive margins. This underreporting is likely caused by the limited sectioning of the tumor sample which we believe can be improved by increased sectioning combined with an automated analysis approach.

## 4. Discussion

As a proof of concept, an automated image analysis tool has been developed which enables whole slide scanning microscopes with automated computational analysis to provide a method for cancer cell detection in a background of debris and noncancerous cells using a novel analysis software to evaluate multiparameter cytologic features. Analysis was performed on prelabeled training data with subsequent validation on an independent testing set, to give a sense of true system performance. The training set was based on an 8 IC slides obtained from resection margins and correlated with confirmed positive or “close” margin on permanent sections. M. E. Ruidíaz and J. Wang-Rodriguez manually identified cells belonging to the class of cancer cells, normal cells, or debris/artifact objects (Figure 1). In evaluation of an LDA classifier of all the parameters, the greatest improvement in classifier performance relative to a 60% baseline (random data) classification accuracy were nuclear color (+23%), cytoplasm color (+15%), shape (+12%), grey (+9%), and local area (+5%) (Figure 2). 

Colorimetric information (RGB and YUV color spaces) from both the nuclear area and the cytoplasm area provides the highest degree classification performance improvement. This is reflected visually with cell-like objects, such as cancer cells and normal cells generally having a dark nuclear area with a lighter cytoplasmic surrounding, while debris/artifacts are more variable and often are within much darker stained surroundings. Furthermore, in normal cells, a well-organized clusters and extremely dark nuclear appearance corresponding to condensed chromatin are observed, while a much larger, diffuse and lighter stained nucleus is present for cancer cells.

Shape information is important primarily in the separation of the debris/artifact class from the biologically derived classes due to cell-like objects having a round nuclear configuration, while generally debris/artifact objects having non-circular configurations. Therefore, a high degree of separation is observed when looking at shape alone. Additionally, it is noted that shape discrimination elucidates a degree of separation between the cancer class and the noncancer cell class. This can be explained due to cancer cells having larger and/or irregularly shaped nuclei than normal cell types.

Identification of specific measurements which best separate each of the classes of objects on an IC specimen was determined by binary classification based recursive feature elimination. Analysis of the top 5 important features for class discrimination revealed the following: (1) cancer cell discrimination is primarily due to the integrated density along with the area. (2) Noncancer cell discrimination is mainly based on the degree of redness of the cell. (3) Debris/artifact discrimination also lies in the red color of the object with less emphasis on the peripheral staining color which is likely due to the lack of cytoplasmic staining of a debris/artifact object. The similar importance of the red color metrics on both (2) noncancer cells and (3) debris/artifact implies a separation is present with noncancer cells having a less red staining while debris/artifact imparts a brighter red profile.

The detection of cancer cells on IC specimens which were judged to be negative (close or clear designation) based on permanent section (A5, A8, and T2) illustrates the utility of this analysis as an intraoperative positive tumor margin detection system and as a permanent section verification tool. However, the failure of both manual and automated analysis to identify cancer cells on margins judged positive by permanent section shows the sampling variation which limits the utility of IC specimens intraoperatively. Improved system performance is expected, with minor software modifications, when applied to various cytology specimens such as fine needle aspiration or core tissue biopsy. It is additionally expected that improvements in IC technology, such as cell adherent coatings, would further increase the number of detected positive specimens during cauterized margin analysis.

## 5. Conclusions

The evolution of high speed slide scanning technology along with the increased ubiquity of computing resources enables a new class of automated analysis tools to discriminate between cancer cells and nonepithelial cells and debris in cytologic evaluation. The system presented in this report to has a 100% agreement between manual and automated analysis, as validated by a small sample set. The study indicates that an automated analysis approach performs well in identification of cancer cells on IC specimens with performance primarily being limited by IC sampling variability due to the heterogeneous nature of specimens with cauterized margins. 

This project demonstrated the proof of concept that an automated system can be successfully utilized to match human interpretation of IC especially when challenged with the difficult situation of identifying rare tumor cells picked up by IC on cauterized surgical margins. The technique is also able to identify some positive margins missed by permanent section allowing for postoperative verification by permanent section analysis. It is expected that other cell based cytologies, such as fine needle aspiration, core biopsy analysis, and even permanent section analysis could be automated with a similar algorithm.

## Figures and Tables

**Table 1:** Slides used for system evaluation consisted of a 16-slide training/testing set.

Subset	Sample number	Diagnosis	Hospital margin status	Location
Training subset	A1	IDC/DCIS	Positive	Deep
	A2	ILC	Positive	Deep
	A3	IDC/ILC/DCIS/LCIS	Positive	Lateral
	A4	IDC	Positive	Deep
	A5	DCIS	Close (<0.1 cm)	Deep
	A6	DCIS	Close (<0.1 cm)	Lateral
	A7	IDC/DCIS	Close (<0.1 cm)	Deep
	A8	IDC/DCIS	Close (<0.1 cm)	Deep

Testing subset	T1	IDC	Close (<0.1 cm)	Deep
	T2	IDC/DCIS/LCIS	Clear (>1 cm)	N/A
	T3	IDC	Clear (>1 cm)	N/A
	T4	IDC/DCIS	Positive	Deep
	T5	IDC/DCIS	Close (<0.1 cm)	Lateral
	T6	IDC/ILC/DCIS/LCIS	Positive	Deep
	T7	IDC/DCIS	Positive	Deep
	T8	ILC/LCIS	Close (<0.1 cm)	Lateral

Pathologic tumor diagnosis (IDC: invasive ductal carcinoma, ILC: invasive lobular carcinoma, DCIS: ductal carcinoma insitu, LCIS: lobular carcinoma insitu) along with permanent section margin status (positive, close, clear) and location (superior, inferior, lateral, medial, anterior, deep) is listed.

**Table 2:** Comparison of permanent section analysis (positive (red), close (orange), clear (green)) to manual and automated imprint cytology analysis (positive, indeterminate, negative). 100% agreement is observed between manual and automated imprint cytology with respect to sample and margin. Specific positive imprint cytology margins are indicated.